# International students’ perceived quality of university health centre services: an exploratory sequential mixed methods study

**DOI:** 10.1017/S1463423624000288

**Published:** 2024-09-20

**Authors:** Putu Ayu Indrayathi, Pande Putu Januraga, Luh Putu Sinthya Ulandari, Putu Erma Pradnyani, Khadija Ramadhan Makame, Nafisa M.K. Elehamer, Soukaina Hilal, Marilynne N. Kirshbaum, Laszlo Robert Kolozsvari

**Affiliations:** 1 Doctoral School of Health Sciences, Faculty of Medicine, University of Debrecen, Debrecen, Hungary; 2 Department of Public Health and Preventive Medicine, Faculty of Medicine, Udayana University, Bali, Indonesia; 3 Health Polytechnic Kartini Bali, Denpasar, Indonesia; 4 School of Health and Medical Sciences, The State University of Zanzibar, Tunguu, Tanzania; 5 Faculty of Public and Environmental Health, University of Khartoum, Khartoum,Sudan; 6 Charles Darwin University, Darwin, Australia; 7 Department of Family and Occupational Medicine, University of Debrecen, Debrecen, Hungary

**Keywords:** international education exchange, perception, quality of healthcare, student health services, universities

## Abstract

**Aim:**

This study aims to investigate international students’ perspectives on service quality and analyse factors contributing to the perceived service quality of the university health centre.

**Background:**

International students are at increased risk of experiencing poor mental health, isolation from families and cultures, language barriers, financial stress and academic pressures. It is important that universities support international students to enable them to complete their degrees and reach their desired level of achievement and performance. One of the supports required by international students is the availability of healthcare services. Therefore, improving the quality of services to meet international students’ requirements, including healthcare services, is essential.

**Methods:**

A three-phase exploratory sequential mixed methods design was used. Phase 1 aims to explore international students’ perceptions of primary healthcare quality by conducting in-depth interviews and focus group discussions. Data were analysed using thematic analysis. Phase 2 is to form questionnaire items based on the results of the qualitative study. The questionnaire is subject to pilot testing to measure validity and reliability. Phase 3 analyses factors influencing international students’ perceived primary healthcare service quality. Multiple regression was used to analyse factors contributing to the perceived service quality of international students.

**Findings:**

The qualitative strand revealed five major themes representing the study participants’ thoughts about the quality of services in the university healthcare context. Perceived quality attributes identified in this study were primarily empathy, equity, effectiveness, efficiency and safety. The quantitative strand found that 35.57% of participants consider the perceived quality of the centre as good. The highest and lowest service quality attributes were related to safety and efficiency, with a score of 21.12 ± 3.58 and 19.57 ± 4.34, respectively. The multiple linear regression analyses showed that PhD students from Health Faculty and Scholarship awardees were significantly associated with the perceived quality of healthcare services. Thus, the university management needs to improve service quality considering the diversity of international students’ socio-demographic characteristics.

## Introduction

The international student population has increased globally. Higher education has created massive transformations and reforms to enable universities to meet the increasingly growing demands for information and knowledge (Wang and Wang 2022; Masai *et al.*, 2021). In Hungary, the internationalisation of higher education has contributed to Hungary’s national economy (Wang and Wang 2022; Yerken and Nguyen Luu 2022). Many international students attend Hungarian universities and colleges to study at all levels (Nemeth *et al.*, 2019; Yerken and Nguyen Luu 2022; Erturk and Nguyen Luu 2022). The number of international students in Hungarian universities and colleges was 38 422 in the 2019/2020 academic year, mostly in Budapest and the universities of Debrecen, Szeged and Pecs (Hungary Today n.d.; Tempus Public Foundation 2018).

It is well-recognised and documented that university students face multiple stressors during their studies, such as academic work, employment, finances, housing and relationships (Mofatteh 2021; Deng *et al.*, 2022). These stresses are often greater and more complex for international students who also need to adjust to differences in culture and language and are far away from familiar supportive relationships such as close family and friends. Studies have shown that international students are at increased risk of experiencing poor mental health compounded by isolation from families and cultures, language barriers, financial stress and academic pressures. Therefore, to enable international students to complete their degrees and reach their desired level of achievement and performance, they will need to be supported by the university in various ways (Umami *et al.*, 2022; Yerken *et al.*, 2022; Erturk and Nguyen Luu,^2022^). Improving the availability of healthcare services would be one of the most important ways to provide much-needed support to international students (Tang *et al.*, 2018).

As one of the host universities in Hungary with international students, the University of Debrecen provides healthcare services for international students through the university health centre (UHC). More than 7000 students worldwide are studying at the University of Debrecen (University of Debrecen 2022). As a global university, the quality of healthcare services and the satisfaction derived from these services by students from different nationalities is one of the most critical issues in the comprehensive quality system for universities (Qasem and Baharun 2011; Aljaberi *et al.*, 2018). Therefore, improving the quality of services to meet international students’ requirements, including healthcare services, is essential (Govender *et al.*, 2014; Lee and Guirguis 2021). Healthcare quality directly correlates to patient satisfaction (Rezaian and Bin Selamat, 2014).

A study stated that patient satisfaction had become one objective of care, representing the patient’s judgement of the quality of healthcare services (Bakan *et al.*, 2014). Furthermore, measuring and understanding patient, caregiver and family experience of healthcare services will provide opportunities for reflection and improvement of care and patient outcomes (Bakan *et al.*, 2014; Gok and Sezen 2013; Hudak *et al.*, 2018). Unfortunately, in Hungary, studies regarding the perceived quality of healthcare and satisfaction mainly focus on patients’ perspectives are still lacking (Brito Fernandes *et al.*, 2019). To the authors’ knowledge, no study has been conducted to investigate the perceived quality of healthcare services among international students in Hungary. Such investigation is expected to contribute to a better understanding of the issues faced by international students concerning healthcare. It will also enable policymakers, managers and providers of healthcare services to enhance the quality of healthcare services. Moreover, problems arising from students’ lack of satisfaction may result in students’ academic and social withdrawal, thus leading to the non-completion of their degree programme (Aljaberi *et al.*, 2018; Julien *et al.*, 2021).

Different conceptual frameworks have been developed to assess healthcare quality and target the general population, and none specifically target international students. As far as we know, this is the first time that this approximation for evaluating primary care has been used in the international student population. The UHC is the primary care provider for international students at the University of Debrecen. The UHC services are free to the students, included in their tuition fee or paid by the Hungarian government (University of Debrecen 2023). Therefore, this study was planned to investigate international students’ perspectives on service quality and analyse factors contributing to the perceived service quality of the UHC. An exploratory sequential mixed methods design (three-phase procedure) addressed these study objectives (Vedel *et al.*, 2018; Fetters *et al.*, 2013; Curry *et al.*, 2013).

## Methods

### Study design

**Figure 1.**
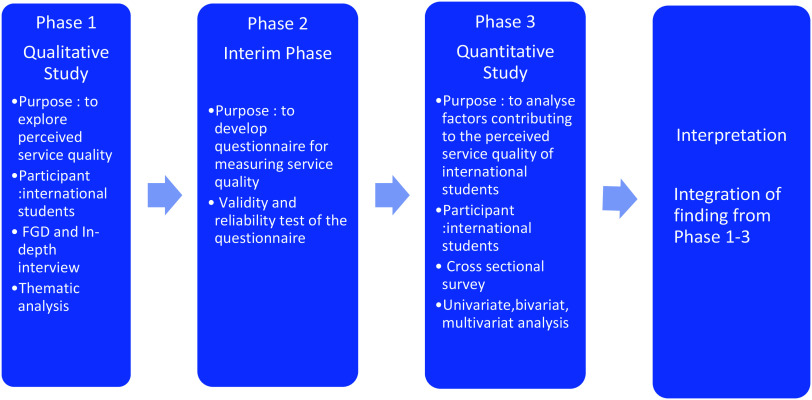
Flowchart of Sequential Mixed Method Design in the study.

Due to the complex interaction between the quality of healthcare service and customer/patient satisfaction and to gain a comprehensive understanding from the international student’s perspective, this research employs an exploratory sequential mixed methods design in which the collection and analysis of qualitative data are conducted before the quantitative data (Curry *et al.*, 2013; Fetters *et al.*, 2013; Vedel *et al.*, 2018). The qualitative study results in the first phase became the basis for developing the questionnaire in the second phase (Fetters *et al.*, 2013). The questionnaire was then tested for its efficacy in the quantitative study. The workflow is presented in Figure 1.

### Setting and population

The study was conducted at the University of Debrecen, Hungary. According to the university website, the university is a home for 29 000 domestic students and more than 7000 international students from 120+ countries studying at all levels of study and faculties (University of Debrecen 2022). Health care is provided by the university for international students. It is coordinated by the Coordinating Centre of the international office, and it is run by the University of Debrecen YouMed, a non-profit organisation. The UHC is on the main campus right behind the Coordinating Centre for International Students. It is open from Monday to Friday from 8am to 4pm. There are four doctors and seven nurses providing services for the students. Students need to call, send an email or book an appointment online before visiting the UHC, but in case of emergency or urgency, they also see same-day walk-in patients (University of Debrecen 2023).

### Phase 1: qualitative study (QUAL strand)

#### Data collection

This strand collected data through focus group discussions (FGDs) and in-depth interviews (IDIs) using interview guidelines. The guideline is developed and modified following WHO guidelines for improving the quality of healthcare services and primary health care (Nejad *et al.*, 2018; Mosadeghrad 2012; World Health Organisation 2018). The interview covered the following key topics: international students’ perception of their health and well-being, health-seeking behaviour and what they mean by quality in healthcare services (Supplementary 1).

The FGDs were held online, and the IDIs were held offline at a place convenient to the participants. The data collection was held from 14 August to 20 September 2022. All interviews were conducted in English, and audio-recorded and detailed notes were taken with the participant’s permission. The objective is to explore the perceived quality of services in the context of primary health care from the perspective of international students. All topics were explored through open questions. The order of questions was flexible according to each informant’s interview response. The interviewers also explored other information outside the interview guide that was interesting and important. The guideline was pre-tested with three international volunteer students of different genders and countries to evaluate the language’s appropriateness and guiding questions.

#### Participants

Participants were recruited using snowball sampling to get the maximum variation of the sampling (different ages, genders, levels of study, nationalities and faculties) to achieve diversity in the participants’ views, experiences and opinions. No predetermined sample size is needed as we used the principle of saturation to reach a proper sample size for this strand, that is, when no more new information or insights can be collected by conducting further interviews (Conneeley 2002; Hesse-Biber 2010). To ensure the anonymity and confidentiality of participants, each participant provided informed consent to participate in the interview and record it.

#### Data analysis

The qualitative data were analysed using thematic analysis, in which the researcher identifies patterned themes. Thematic analysis steps include interview transcription, familiarisation of data, open coding of the entire data, axial coding (identifying the association of codes and integrating associated codes into a thematic category) and selective coding (selecting and integrating categories into main themes) (Braun and Clarke 2006). Strategies to increase the trustworthiness of the research findings included data source triangulation with the use of FGD and IDI for data collection (Carter *et al.*, 2014) and peer debriefing with experts in primary healthcare and quality services (Curry *et al.*, 2013; Vedel *et al.*, 2018). NVivo 12 Plus software was utilised in the data analysis process.

### Phase 2: interim phase

In the interim phase, the qualitative findings were used to develop a questionnaire to be tested in the quantitative study. The questionnaire was refined after consultation with primary care physicians and healthcare quality experts who provided input regarding the content and delivery formats. The questionnaire then underwent a pilot test on 30 international students from various backgrounds, including gender, age, nationality and level of study. The validity and reliability of the questionnaire were analysed, and revisions were made accordingly. The results of validity and reliability tests with the Pearson correlation statistical test (*r* count > *r* table or ir-cor more than 0.3) and Cronbach alpha (>0.6) mean that the instrument used is valid and reliable and ready to distribute.

### Phase 3: quantitative study (QUAN strand)

#### Data collection

The research then continued with a quantitative strand using a cross-sectional survey. An analytic, quantitative study was conducted to determine what factors determine international students’ perceived quality of services. Data were collected using a structured questionnaire for international students. A questionnaire was developed based on findings from the prior qualitative study, which explored international students’ perspectives on perceived service quality. The questionnaire consisted of three (3) sections with 37 questions. The first section includes 10 questions about socio-demographic profiles, including gender, age, nationality, marital status, level of study, faculty, student’s status, sponsorship, religion, the last visit to UHC service and the frequency of visiting the UHC service. The second section consists of 25 items incorporating the 5 main themes from the qualitative study result. The last section consists of an open question asking comments about the quality of care the university provides for international students (Supplementary 2). The questions in the questionnaire were scored based on the Likert scale from 1 (total disagreement) to 5 (total agreement).

The cross-sectional survey was developed as an online survey using Google Forms and offline surveys.

The online survey was distributed to international students through social media platforms like Facebook, Instagram and WhatsApp. At the same time, the offline survey was distributed through the UHC and researchers’ networks. Data collection was carried out from 15 November 2022 to 8 March 2023. The combination of online and offline was chosen to minimise bias due to the standard challenge of online surveys, such as low response rate, unequal participant distribution and participant fraud (Rice *et al.*, 2017). The onsite requirement was conducted to minimise it, although recall bias is expected in both online and onsite questionnaires (Rice *et al.*, 2017; Aljaffary *et al.*, 2022).

#### Participants

The calculations with the Raosoft Calculator show that the minimum number of samples required is 363 respondents. The criteria in this study are international students in the University of Debrecen aged ≥ 18 years, having active student status from the University of Debrecen, living in Debrecen for at least 3 (three) months, having visited or used the UHC (GP clinic) at least 1 (one) time. The study participants were international students at all levels at the University of Debrecen and were recruited with the convenience sampling method.

#### Data analysis

All data were edited and cleaned for analysis. Descriptive statistics were used to obtain variable distributions (ie, frequencies, percentages, means and SD). We applied a bivariate linear regression test to determine crude associations between independent and dependent variables; we nominated candidate variables with *P*-values < 0.25. A multiple linear regression analysis was performed to determine which independent variables were associated with the dependent variable. Results were considered significant based on *P*-values < 0.05. All data analyses were conducted using STATA 12.0.

## Results

### Qualitative results

A total of 16 international students were interviewed, and the average duration of an interview was approximately 45–60 min. Table 1 describes participants involved in the qualitative strand. As shown, participants share the same number in terms of gender, mostly from Asia, pursuing a doctoral degree, and from non-health-related faculties.

**Table 1.** Socio-demographic characteristics of qualitative study participants

No	Description	Total
1	Age (mean, range)	30.05, 23–42
2	Gender	
	Male	8
	Female	8
3	Level of study	
	Master	7
	Doctoral	9
4	Nationality	
	Asia	12
	Africa	3
	America	1
5	Faculty	
	Health	7
	Non-health	9

The analysis from FGDs and IDIs found five major themes that represented the study participants’ thoughts about the quality of services in the context of primary health care. According to international students, the UHC should provide five main attributes of quality healthcare services. Healthcare service attributes identified in this study were primarily empathy, equity, effectiveness, efficiency and safety (4ES).

**Figure 2.**
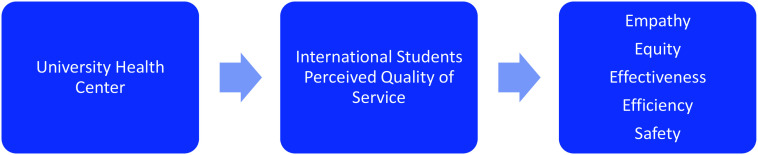
Thematic framework of perceived quality services.

Figure 2 shows a model of quality dimensions in the UHC (the 4ES’s model). Each of the dimensions can be defined as follows: empathy, caring and individualised attention that the healthcare provider provides to its patients; equity, treating every patient the same; effectiveness, health centres provide evidence-based healthcare services; efficiency, health centres can maximise the benefit of available resources; and safety, the health centre and health workers could avoid harm to patients and reduce the risk of unnecessary harm. Table 2 shows quotes of evidence to support the finding of the qualitative strand.

**Table 2.** Examples of how international students perceive the quality of service

Theme	Sub-themes	Quotation
Empathy	Treating student nicely Willingness to listen more Comfortable consultation	*‘Like, for example, they treat me nice, they smile to show.showing genuine smile’. (IDI_M_3)* *‘.well in my opinion they have to show a little bit more empathy. we have no family and far from home and listening us more’. (FGD_1_M)* *‘.to some extents I feel not comfortable to discuss about my sickness. I am not feeling comfortable during consultation seems to me they looks bit judgemental.maybe just my feeling.but really felt not comfortable’. (IDI_F_5)*
Equity	Treating with dignity and compassion Treating patient the same	*‘.after get the services, I just hope that staff will keep treat Us with dignity and compassion’. (IDI_F_1)* *‘.just hoping we can get the same treat as domestic students’. (FGD_3_M)*
Effectiveness	Effective communication Health improvement	*‘So like we sometimes with the ability to speak in English and so we are very clueless. like what should we go, what should we do, what should they mean?’* (IDI_F_7) *‘feels much better after get examination…the GP really helpful.the lady doctor quite nice’.*
Efficiency	Difficulty to get an appointment Lack of medical staff	*‘sometimes like somehow difficult to get service from them, because usually not easy to get appointment’*. (FGD_2_M) *“.only few doctor available for international students.causing long waiting time.I cannot waiting long because I will have a class’. (FGD_1_M)”*
Safety	Explaining the purpose of the test and treatment Safe patient experience	*‘the people go get treatment and say, oh your fine. only pain and he(GP) didn’t explain in detail.maybe because of language barrier’. (FGD_1_M)* *‘.for me it is important to feel safe when seeking care.cleanliness of the place and the tools use for example’. (IDI_9_F)*

### Quantitative results

In the quantitative strand, 437 international students participated in the cross-sectional survey, but only 402 (91.99%) were analysed. Thirty-five responses were excluded because participants did not meet the inclusion criteria or submitted incomplete answers. Table 3 shows their socio-demographic characteristics.

**Table 3.** Socio-demographic characteristics of quantitative study participants

Variables	*n* (%)	Min	Max	Mean (SD)	Median (IQR)
Age	402	18	50	23.72 (5.51)	22 (6)
Gender					
Male	196 (48.76)				
Female	206 (51.24)				
Nationality					
America	20 (4.98)				
Africa	152 (37.81)				
Asia	209 (51.99)				
Europe	21 (5.22)				
Level of study					
Bachelor	187 (46.52)				
Master	73 (18.16)				
PhD	53 (13.18)				
1 tier degree	64 (15.92)				
Others	25 (6.22)				
Marital status					
Single	317 (78.86)				
In a relationship	51 (12.69)				
Married	34 (8.45)				
Faculty					
Non-health	223 (55.47)				
Health	179 (44.53)				
Student status					
First year	165 (41.04)				
Second years	101 (25.12)				
Third years	60 (14.93)				
Fourth years	26 (6.47)				
Others	50 (12.44)				
Sponsorship					
Self-payment	204 (50.75)				
Scholarship	198 (49.25)				
Religion					
Christian	149 (37.06)				
Muslim	173 (43.03)				
Others	30 (7.46)				
No religion	50 (12.44)				
Last visit to the UHC					
Less than 1 month	160 (39.80)				
Between 1 and 2 months	90 (22.39)				
More than 3 months	152 (37.81)				
Perceived quality	402	25	125	102.22 (17.51)	103 (26)

UHC = university health centre; IQR = interquartile range.

As shown, the mean age of respondents was 23.72 (5.51), ranging from 18 to 50 years. 51.25% of respondents are female, and 48.76% are male. The majority of the samples are from Asia (51.99%), followed by students coming from Africa (37.81%), Europe (5.22%) and America (4.98%). Furthermore, most of them are bachelor students (46.52%), unmarried (78.86%), from non-health-related faculties (55.47%), first-year students (41.04%), self-payment (50.75%) and Moslem (43.03%), and 39.80% of participants said that their last visit to the health centre was less than one month.

The perception dimensions of international students were composed of 5 attributes based on the 25 statements from the qualitative study. The mean score of the perceived quality attributes is shown in Table 4.

**Table 4.** The mean score of the perceived quality attribute

Quality attributes	N	Min	Max	Mean (SD)	Med (IQR)
Empathy	402	5	25	20.96 (3.70)	21 (6)
Equity	402	5	25	20.63 (3.97)	21 (7)
Effectiveness	402	5	25	19.94 (4.07)	20 (6)
Efficiency	402	5	25	19.57 (4.34)	20 (6)
Safety	402	5	25	21.12 (3.58)	20 (6)

IQR = interquartile range.

The highest and lowest service quality attributes were related to safety and efficiency, with a score of 21.12 (3.58) and 19.57 (4.34), respectively. Furthermore, internal consistency (Cronbach’s alpha) was initially calculated for the quality attributes to determine the magnitude of international students’ perceived quality of the UHC services. The result of Cronbach alpha measurement value is 0.913. All 25 attributes to measure perceived quality had a maximum score of 125 and a minimum of 25. Based on the mean percentage score, 143 (35.6%) of the study participants rated the perceived quality of the UHC as good. Table 5 shows the result of both bivariate and multiple analyses. The bivariate analysis showed that several factors were statistically associated with the perceived quality of services. It was then followed with multivariate analysis including candidate variables with bivariate analysis *P*-values < 0.25.

**Table 5.** Factors affecting perceived service quality of international students

Variable	Bivariate analysis				Multivariate analysis			
	95% CI			*P*-value	95% CI			*P*-value
	B	Lower	Upper		B	Lower	Upper	
Age	−0.29	−0.61	0.02	0.067	−0.24	−0.58	0.10	0.168
Gender								
Male	ref							
Female	−1.68	−5.11	1.75	0.337				
Nationality								
America	ref				ref			
Africa	5.56	−2.64	13.76	0.183	5.85	−2.27	13.97	0.157
Asia	4.88	−3.19	12.95	0.235	5.68	−2.3	13.66	0.162
Europe	3.55	−7.22	14.33	0.517	3.98	−6.61	14.59	0.460
Level of study								
Bachelor	ref				ref			
Master	−2.09	−6.79	2.61	0.382	−1.2	−5.97	3.58	0.623
PhD	−8.16	−13.47	−2.86	0.003	−5.91	−11.58	−0.23	0.041
1 tier	−5.74	−10.67	−0.81	0.023	−3.42	−9.03	2.19	0.231
Others	−5.79	−13.05	1.45	0.117	−5.69	−13.2	1.82	0.137
Marital status								
Single	ref				ref			
In a relationship	2.66	−2.52	7.85	0.313	3.67	−1.66	9.00	0.177
Married	−4.83	−11.02	1.37	0.127	−0.73	−9.72	8.25	0.873
Faculty								
Non-health	ref				ref			
Health	−4.81	−8.23	−1.38	0.006	−5.38	−9.44	−1.32	0.010
Student status								
First year	ref				ref			
Second years	−4.63	−8.97	−0.29	0.037	−3.57	−8.15	0.99	0.125
Third years	−0.51	−5.69	4.67	0.847	−0.02	−5.29	5.24	0.994
Fourth years	−2.63	−9.89	4.62	0.476	−1.05	−8.44	6.33	0.779
Others	0.34	−5.2	5.89	0.902	4.60	−1.66	10.88	0.149
Sponsorship								
Self-payment	ref				ref			
Scholarship	−2.88	−6.31	0.54	0.099	−4.49	−8.58	−0.39	0.032
Religion								
Christian	ref							
Muslim	−0.15	−4.01	3.71	0.941				
Others	0.38	−6.53	7.29	0.914				
No religion	0.04	−5.6	5.69	0.988				
Last visit time								
Less than 1 month	ref				ref			
Between 1 and 2 months	−0.67	−5.21	3.86	0.771	−0.08	−4.67	4.50	0.972
More than 3 months	−2.61	−6.51	1.29	0.190	−0.92	−5.34	3.52	0.686

CI = confidence interval.

The multiple linear regression analysis showed that PhD students from the health faculty and scholarship awardees were significantly associated with the perceived quality of healthcare services the UHC provided. It appears that the PhD level is the level of study that most significantly affects the perceived quality of healthcare services the UHC provides compared with the bachelor level, but this effect is negative, meaning that the PhD level of study decreases the perceived quality of healthcare services the UHC provides compared with the bachelor level. From the type of faculty, health-related faculties decrease the perceived quality of healthcare services the UHC provides compared with non-health faculties. Additionally, scholarship awardee students have less experience in the perceived quality of healthcare services provided by the UHC compared with self-funded students.

## Discussion

The present study sheds light on an important yet under-investigated topic of the quality of healthcare service for international students in Hungary. Understanding patient experience is crucial for healthcare organisations. This is because patient experience is widely recognised as one of the key elements of quality within healthcare organisations, especially in enhancing competitive growth strategy (Bellio and Buccoliero 2021; Govender *et al.*, 2014). Furthermore, providing patient-centred care has become one of the basic requirements of good quality care (Bakan *et al.*, 2014; Bellio and Buccoliero 2021). Several studies suggest that positive patient experience improves health outcomes, patient loyalty and satisfaction (Murante *et al.*, 2014; Bellio and Buccoliero 2021). It is, therefore, important to investigate attributes that contribute to the perceived quality of international students because they come from different backgrounds and cultural diversity. The qualitative study found five major attributes related to the perceived quality of international students who accessed UHC: empathy, equity, effectiveness, efficiency and safety (4ES). This qualitative study’s findings might supplement quality indicators in the healthcare sectors, which mainly focus on the general population, such as SERVQUAL, HEALTHQUAL, PubHosQual, HospitalQual models, Primary Care Assessment Tool, etc. (Endeshaw 2021; Lévesque *et al.*, 2012). Furthermore, it can provide information on meeting international students’ expectations of service quality in healthcare.

This study found that the highest score for the quality attribute in UHC services is safety. This means that according to international students, safety standards and procedures at the UHC meet their expectations. Based on the six pillars of high-quality care, safety refers to high-quality care to avoid preventable patient harm (Hannawa *et al.*, 2022). The UHC seems committed to putting patient safety at a high standard for international students. This commitment supports the WHO agenda that primary healthcare providers should prioritise patient safety (Aljaffary *et al.*, 2022; Alameddine *et al.*, 2015). This is because patient safety is a pivotal attribute of healthcare quality in primary health care (Aljaffary *et al.*, 2022; Ghobashi *et al.*, 2014). The quantitative study also found that the UHC’s service efficiency got the lowest score among international students. It has been suggested that international students perceive that efficiency attributes are not meeting the students’ expectations, especially in terms of finding appointments to get care from the GP. The efficiency of healthcare delivery is related to the supply and delivery of timely services to the vulnerable population (Tormusa and Mogom Idom 2016). Based on qualitative study findings, the international students perceived that finding an appointment to gain care from GPs in the UHC was relatively difficult and resulted in experiencing a relatively long waiting time. The finding aligns with the results of several studies, which found that patients were least satisfied with long waiting times (Singh and Dixit 2020; Ogunnowo *et al.*, 2015; Soneta *et al.*, 2021). Such delays might prevent students from seeking care, especially when they have academic work and are committed to their studies (Collins 2001; Carmack *et al.*, 2016; Tang *et al.*, 2018). As a result, it may lead students to do self-care or seek out alternative remedies (Carmack *et al.*, 2016; Soneta *et al.*, 2021; Lee and Guirguis 2021; Tang *et al.*, 2018)

Our study also found that PhD students were significantly associated with the perceived quality of the UHC’s healthcare services. This might occur because PhD students study longer, and the academic burden is greater and more intensive than bachelor’s and master’s degrees. A study suggests that the high academic burden of a PhD student might increase the development of a common psychiatric disorder, especially depression (Levecque *et al.*, 2017). This may lead to the increasing demand for healthcare services for PhD students. Students from health-related faculties were also significantly associated with the perceived quality of healthcare services the UHC provided. The potential reason might be that their healthcare awareness is relatively higher and they better understand healthcare services than their counterpart from non-healthcare faculties (Patel *et al.*, 2017).

Furthermore, international students holding scholarships were also significantly associated with the perceived quality of healthcare services. Students with Hungarian Governmental Scholarships are entitled to have a Hungarian Social Security card. It allowed those international students with scholarships to benefit from healthcare services similar to local Hungarian (University of Debrecen 2023; Egeszsegvonal 2022). On the other hand, self-sponsorship students are offered compulsory combined health and travel insurance, which is included in the tuition fee (University of Debrecen, 2023). These different types of health insurance may contribute to the perceived quality of international students. Scholarship students with Hungarian Social Security cards have a wide range of choices to get access to healthcare services, as this card is acceptable for all Hungarian healthcare services that the Hungarian-insured citizens receive.

## Limitations of the study

While this study produced valuable findings, there were also some limitations. The present study’s main limitation was that the qualitative study’s findings regarding attributes to the perceived quality of international students could not be generalised as it is only sourced from one university in Hungary. Furthermore, convenience sampling rather than randomisation was used in a single cohort, where the responses may be biased and less reliable. The investigation in this study was exclusive to the perceived quality of healthcare service from the international student’s perspective. Therefore, future research could also include the perspective of healthcare workers and management to gain deeper insights into the perceived quality of healthcare services.

## Conclusions

The study showed that five attributes might be considered to improve healthcare services for international students. Patient safety is the predominant domain of international students’ perceived quality in the UHC. Furthermore, being PhD students, studying in health-related faculties and being scholarship recipients predicted international students’ perceived quality. Therefore, it is important for the UHC management and the university to improve service quality. The improvement needs to consider attending responsibly and effectively to the diversity of international students’ socio-demographic characteristics. This would help address the many difficulties international students face, most prominently by primary care services and healthcare structures that differ greatly due to social, historical, economic and cultural factors.

## Supporting information

Indrayathi et al. supplementary material 1 Indrayathi et al. supplementary material

Indrayathi et al. supplementary material 2 Indrayathi et al. supplementary material

## Supplementary material

To view supplementary material for this article, please visit https://doi.org/10.1017/S1463423624000288.
