# Supplementary material for: International students’ perceived quality of university health centre services: an exploratory sequential mixed methods study
Source: Prim Health Care Res Dev. 2024 Sep 20;25:e39. doi: 10.1017/S1463423624000288 (PMC11464857; doi:10.1017/S1463423624000288)
Supplement: Indrayathi et al. supplementary material 1 — Indrayathi et al. supplementary material [file S1463423624000288sup001.docx]

Interview Guide for International Students

**“An Assessment of International Students’ Perceived Quality of University Health Centre Services”**

Introduction

Thank you for your participation in this study. We are interested in finding out what international students at the University of Debrecen feel and think about their health and perception of the service provided by the University Health Center, particularly what problems and difficulties you or other international students may have had with healthcare services. This interview will last for about 45 – 60 minutes. Do you have any questions before we proceed?

Health and wellbeing and health-seeking behaviour

| Leading Question | Probing Questions |
| --- | --- |
| Can you tell us what is thought about health and well-being as international students? | Probe:   1. What kinds of words, description or feelings come to mind? 2. What kinds of feeling are associated with health and wellbeing? 3. What things become difficult when your health and wellbeing are low/compromised? |
| Have you ever experiencing health related problems during your stay and studying in Debrecen? | Probe :   1. What kind of problem? 2. How you manage it? 3. Is it manageable? 4. Who do you normally see if you health related problem? |

Student experience on visiting or being treated at the University Health Center

| Leading Question | Probing Questions |
| --- | --- |
| Can you please what is your experience when visiting or being treated at the university health center? | Probe:   1. What happened? 2. Was it for acute or ongoing health issues? |
| In general, how would you describe your experience in accessing the university health center? | Probe :  Overall satisfaction? if not happy, explore in depth what is the reason |
| What is your thought about being treated by the UHC staff? | Probe :   1. Do you feel safe and trust the health workers? 2. Do the health care workers show their empathy and treat patients fairly? 3. Do you have enough time to consult about your condition with health care workers in the centre? 4. How about waiting time to get care? Is it on time? |
| Would you share with me, what is the difference of health care system here and in your country | Probe:   1. Is there any specific differences? 2. What about your health insurance? Is this enough to cover the financial costs of seeking medical services in Debrecen? Do you know what is covered and not covered by health insurance? |

Student perspective on healthcare service quality

| Leading Question | Probing Questions |
| --- | --- |
| In your opinion, how would you describe about healthcare service quality | Probe :   1. What are the best words to describe good services? 2. Why is it important in good services?   Note : keep focusing to explore any option made by students about healthcare quality of services |
| What is your opinion about the quality of care provided to you in the UHC? | Probe   1. Were there any concerns, challenges, or potential conflicts for you with the service delivery? 2. Would you prefer to be treated elsewhere?   Note : explore the reason for any option made by the students! |
| Based on your personal experiences, how can the UHC improve the quality of services for international students? | Probe:   1. Improving service delivery model and management plan? 2. Do you mind sharing some positive or negative of being treated by the healthcare worker in the UHC? 3. What would you like to see changed or improved regarding medical services to international students? What services or organisations have you sought help from? |

Closing

Thank you very much for your time and your participation in this study. Before I end the interview, is there any question you would like to ask?
