# Supplementary material for: International students’ perceived quality of university health centre services: an exploratory sequential mixed methods study
Source: Prim Health Care Res Dev. 2024 Sep 20;25:e39. doi: 10.1017/S1463423624000288 (PMC11464857; doi:10.1017/S1463423624000288)
Supplement: Indrayathi et al. supplementary material 2 — Indrayathi et al. supplementary material [file S1463423624000288sup002.docx]

**Participant information**

The Department of Family Medicine and Occupational Health, Faculty of Medicine, University of Debrecen, Hungary, is currently conducting research: **Assessment of International Student health, well-being, and perceived service quality of primary care.** We know that international students face many challenges in their studies and adjusting to life in Hungary. These challenges include language difficulties, financial problems, homesickness, illness, accommodation issues, issues with employment, lack of emotional and social support and finding friends that might affect the health and health-seeking behaviour.

We are interested in hearing about the kinds of challenges faced by the University of Debrecen students in Hungary so that we can better understand your experiences and improve services that will assist students in improving their health and well-being while studying in Hungary. To collect information about what would make a difference to your studies and quality of life, researchers from the University of Debrecen will hold a survey. Please be reassured that your views will be treated as STRICTLY CONFIDENTIAL, and no students will be identified by name. That is, all participants in the research will remain ANONYMOUS. Furthermore, the research has received ETHICS APPROVAL from the University of Debrecen, which means that: involvement in this research is entirely voluntary, it will be conducted in a safe and supportive environment, the information collected will be used for research purposes only, and participation or non-participation will not affect your studies or access to services.

Many thanks for your consideration.

**Consent Form**

I now consent to participate in the research project and understand the Information Sheet on the above project. I understand that I may not benefit directly from this research. I understand that while information gained in the study may be published, I will not be identified, and all individual information will remain confidential. I consent to be involved in this project.

Signature: ..............................................................................................

Date: ......................................................................................................

1. **Demographic Data**

1.Gender (please tick one)

Female

Male

Others: ……………………………

2. Age: ……………………………

3. Nationality: …………………………….

4. Marital Status (please tick one)

Single

In a relationship

Married

Divorced

Others

5. Level of study: (please tick one)

Bachelor

Master

PhD

1 Tier Degree (Medical and Dentistry)

Others: …………………..

6. Faculty :

Healthcare-related (please specify:………………………………..)

Non-Health care related (please specify:………………………………..)

7. Student’s Status: (please tick one)

First Year

Second Year

Third Year

Fourth Year

Others

8. Sponsorship :

Scholarship (please specify: ………………………………….)

Self sponsor

9. Which, if any, of the following best describes your religion

Christian

Muslim

Jewish

Buddhist

Hindu

Sikh

No Religion

Others:……………………………………..

10. Last visit time to University GP’s service : (please tick one)

Less than 1 month

Between1-2 months,

More than 3 months

11. How frequently are you visit University GP’s service? (please tick one)

1 time per month

2-3 times per month

Others : …………………………………..

1. **Performance of University Health Care [This part is to describe your experience while using the service provided by the University Health Center (GP office) in the University of Debrecen]**

**Please tick (√ ) to the statement strongly agree, agree, undecided, disagree, or strongly disagree.**

|  | **Statement** | **What do you feel?** | | | | |
| --- | --- | --- | --- | --- | --- | --- |
|  |  | **Strongly Agree** | **Agree** | **Undecided** | **Disagree** | **Strongly Disagree** |
| **S1** | **Empathy** | | | | | |
| S11 | Health workers listening to you |  |  |  |  |  |
| S12 | Health workers help you to feel well so that you can perform your normal daily activities |  |  |  |  |  |
| S13 | Health care workers tell you what you want to know about your symptoms and illness |  |  |  |  |  |
| S14 | Health care workers make you feel comfortable during consultation |  |  |  |  |  |
| S15 | Health care workers involving you in decisions about your medical care |  |  |  |  |  |
| **S2** | **Equity** | | | | | |
| S21 | Health workers treating every patient exactly the same |  |  |  |  |  |
| S22 | Health care workers treated with dignity and compassion |  |  |  |  |  |
| S23 | Health care workers are always willing to help |  |  |  |  |  |
| S24 | Health care services are affordable |  |  |  |  |  |
| S25 | Comprehensive service available to all |  |  |  |  |  |
| **S3** | **Effectiveness** | | | | | |
| S31 | Health workers use effective English in providing care |  |  |  |  |  |
| S32 | Health workers knowing what s/he had done or told you during contacts |  |  |  |  |  |
| S33 | Quick relief of your symptoms after examination |  |  |  |  |  |
| S34 | A visit to the doctor usually results in an improvement in health |  |  |  |  |  |
| S35 | My physical and mental state improved after the visit to the doctor |  |  |  |  |  |
| **S4** | **Efficiency** | | | | | |
| S41 | Getting an appointment to suit you |  |  |  |  |  |
| S42 | Getting service through digital services |  |  |  |  |  |
| S43 | Waiting time for examination fast |  |  |  |  |  |
| S44 | Health workers provide service competently |  |  |  |  |  |
| S45 | Overall service time in line with expectation |  |  |  |  |  |
| **S5** | **Safety** | | | | | |
| S51 | Create a safe patient experience |  |  |  |  |  |
| S52 | Explaining the purpose of tests and treatments |  |  |  |  |  |
| S53 | The center insists on error free record |  |  |  |  |  |
| S54 | Medical devices use in the center |  |  |  |  |  |
| S55 | Keeping your records and data confidential |  |  |  |  |  |

1. **Open Questions**
2. Do you have any other comments about the quality of care the University Health Center provides?
3. What other kinds of services do university should provide for international students?

------- **Thank you for your participation** -------
